# A Numerical Method for Applying Cohesive Stress on Fracture Process Zone in Concrete Using Nonlinear Spring Element

**DOI:** 10.3390/ma15031251

**Published:** 2022-02-08

**Authors:** Zhuheng Li

**Affiliations:** State Key Laboratory of Coastal and Offshore Engineering, Dalian University of Technology, Dalian 116024, China; s201051122@mail.dlut.edu.cn; Tel.: +86-137-9519-3001

**Keywords:** cohesion on FPZ, iterative approach, nonlinear spring element, displacement control, P-delta curve, P-CMOD curve, FPZ length, K_R_ curve

## Abstract

Aiming at the numerical simulation of the entire crack propagation process in concrete, a numerical method is proposed, in which cohesive stress on the fracture process zone (FPZ) is simulated and applied by a nonlinear spring element. Using displacement control, the cohesive stress values on the FPZ are obtained from solving a system of nonlinear equations through an iterative process. According to a crack propagation criterion based on initial fracture toughness, the approach adds the spring elements to finite element analysis when simulating mode I crack propagation in standard three-point bending notched concrete beams with different strengths, initial crack ratios ${(a}_{0}/D)$, and depths (D). The simulated load versus displacement (P-Delta) curves are performed to recalculate the fracture energy and verify the accuracy of cohesion in the proposed method. The simulated load versus crack mouth opening displacement (P-CMOD) curves are consistent with the previous experimental results. Subsequently, the variations of the FPZ length and the crack extension resistance ($K_{R})$ curves are studied according to the proposed iterative approach. Compared with the existing methods using a noniterative process, the iterative approach generates a larger maximum FPZ length and $K_{R}$ curve where the FPZ length is mainly determined by the fracture energy, tensile strength, and geometry shape of the beam, and the $K_{R}$ curve is primarily determined by the fracture energy and FPZ length. The significant differences in numerical results indicate that the applying cohesion is essential in numerical simulation. It is reasonable to conclude that the proposed nonlinear spring element is more applicable and practical in the numerical simulation of the concrete mode I crack propagation process by improving the accuracy of the cohesion applied on the FPZ.

## 1. Introduction

It is well known that concrete is a quasi-brittle material. The cracking behavior of notched concrete beams is influenced by size effects and environmental conditions. Fracture tests have been conducted to investigate the effects of these factors [1,2]. In addition, scholars have proposed different fracture criterions to determine the instability of cracks in concrete, such as the maximum circumferential stress criterion [3], the maximum energy release rate criterion [4], and the double-K fracture criterion [5,6]. Specifically, the double-K fracture criterion uses crack initial fracture toughness and unstable fracture toughness to determine the initiation and instability of concrete fracture. Subsequently, researchers have studied all steps of crack development based on crack propagation criterions, which could reflect the association between the propagation force at the crack tip and the material’s own resistance during crack propagation.

To describe the strain softening behavior of concrete fracture, the concept of fracture process zone (FPZ) was established. By considering attractive atomic forces, Barenblatt [7,8] introduced FPZ, which was defined as a confined area near the crack tip. Furthermore, a different theory was proposed by Dugdale [9], which stated that the stress was equivalent to the yield strength of the material that acted across the crack within the plastic zone near the crack tip. This theory is applicable to ductile metal materials. Later, Hillerborg et al. [10] presented a fictitious crack model to represent the FPZ of quasi-brittle materials, where cohesions were distributed on both sides of the fictitious crack according to the settled softening constitutive relationship. The softening constitutive relationship of concrete, such as linear [10], bilinear [11], and exponential curves [12], described the characteristics of cohesive force on the FPZ. Fracture energy $G_{f}$ represented the envelope area under the softening constitutive curve and concluded that it was a necessary parameter in the numerical analysis of the concrete fracture process. It could be determined through experiments, such as direct tensile tests [13] or three-point bending beam tests [14].

The fictitious crack model has been widely adopted and applied in the crack propagation process simulation. Based on the fictitious crack model proposed by Hillerborg [10], Gerstle and Xie [15] simulated the crack propagation process according to the maximum tensile strength criterion by using a linear softening constitutive relationship. Carpinteri and Massabó [16] introduced a crack propagation criterion characterized by the stress intensity factor for the mode I fracture of cement-based materials, which can be expressed as Equation (1):

(1) $$K_{I}^{P}-K_{I}^{\sigma }=0,$$

where $K_{I}^{P}$ represents the stress intensity factor generated by external force, and $K_{I}^{\sigma }$ represents the stress intensity factor generated by cohesion. The criterion stated that when the crack was in a critical state, the difference between the stress intensity factor caused by external force and cohesion was zero at the crack tip. Ooi and Yang [17,18], Yang and Deeks [19], and Moës and Belytschko [20] applied this criterion to simulate the mode I and mix-mode fracture of reinforced concrete and plain concrete. However, concrete is a quasi-brittle material, and $K_{I}^{P}{-K}_{I}^{\sigma }$ is supposed to be a finite value. Based on this, Wu et al. [21] proposed an intensity-factor-based fracture propagation criterion where the difference between the stress intensity factor at the crack tip generated from external load and cohesion was greater than the initiation fracture toughness ($K_{\mathrm{Ic}}^{\mathrm{ini}}$), and crack started propagating. The propagation criterion can be shown as Equation (2):

(2) $$K_{I}^{P}{-K}_{I}^{\sigma }\geq K_{\mathrm{Ic}}^{\mathrm{ini}},$$

This modified criterion is recently widely used in simulating the entire process of crack propagation, such as: three-point bending beams [21], infinite slab [22], concrete gravity dam model [23], concrete mode I [24], modes I–II [25] fracture of different strengths, and bimaterial interface crack propagation [26]. Meanwhile, numerical methods for calculating the FPZ length and $K_{R}$ curve are developed [21,22].

The main difference of the above literature is the utilization of different fracture propagation criterions in finite element numerical simulation to obtain the entire process of fracture propagation. What needs to be emphasized is that whichever fracture propagation criterion is used for numerical simulation, criterions are always based on the fictitious crack model, and the cohesion applied in the fictitious crack is an important link to affect the numerical simulations.

**Figure 1:**
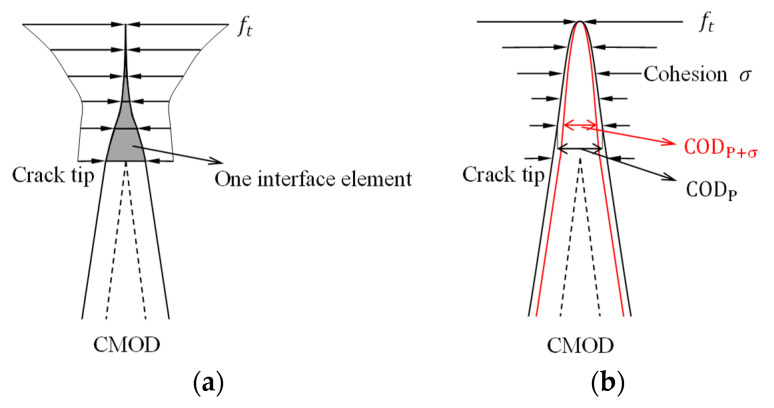
Methods to apply cohesion on the FPZ: (**a**) insert interface element into FPZ, (**b**) directly apply cohesion on FPZ.

There are mainly two methods to apply cohesion in fictitious crack during crack propagation. The first method is to add the interfacial elements to characterize the cohesion in a separate crack, as shown in Figure 1a. Ingraffea et al. [27,28,29] used six-node interface elements to characterize fictitious crack for the analysis of complex fracture, but the nonlinear calculation efficiency was low. Swenson [30] used the six-node interface elements to simulate the dynamic crack propagation process. In the analysis of Bocca et al. [31] and Gerstle and Xie [15], a four-node linear displacement interface element was used to characterize the cohesion in the fictitious crack. Moreover, none of the above-mentioned finite element models can calculate or apply stress intensity factors.

The second method to apply cohesive stress is considering the calculation results of a finite element and directly applying cohesion to the corresponding crack surface nodes as a boundary condition [21,22,23,24,25,26], as shown in Figure 1b. It is noticeable that the cohesion applied in this process is not accurate enough. Crack opening displacement (COD) is obtained from external load, P, rather than the superposition of external load and the cohesion, P + σ, denoted by ${\mathrm{COD}}_{P+\sigma }{\;<\;\mathrm{COD}}_{P}$, where ${\mathrm{COD}}_{P+\sigma }$ is COD induced by P + σ and ${\mathrm{COD}}_{P}$ is COD induced by P, so that the corresponding cohesion relationship is $\sigma_{P+\sigma }{\;>\;\sigma }_{P}$, where $\sigma_{P+\sigma }$ is a recalculated cohesion under P + σ and $\sigma_{P}$ is a cohesion calculated from P based on the softening law, which indicates that the cohesion applied on a crack surface according to this method is less than the theoretical value. To obtain a more realistic cohesive stress value, it is plausible to use the multiple iterations of ${\mathrm{COD}}_{P+\sigma }$ and σ. However, it is incorrect to solve the nonlinear problem, and this iterative method often gives a nonconvergent result. Although the latter method considered the initial fracture toughness of concrete and used it as a control parameter for a crack propagation criterion, cohesion on the FPZ has not been applied accurately in the past research [21,22,23,24,25,26]. Therefore, other methods need to be explored to effectively solve the nonlinear problem.

In this paper, a numerical method is proposed, in which cohesive stress on FPZ in concrete is simulated and applied by a nonlinear spring element. The crack propagation criterion based on initial fracture toughness, which considers the stress singularity at the crack tip, is used. The proposed method improves the accuracy of cohesion applied on the FPZ and optimizes the numerical simulation process through displacement control. Subsequently, the P-delta curves, the P-CMOD curves, the variation of the FPZ length, and the $K_{R}$ curves are obtained. The simulated P-delta curves are performed to recalculate fracture energy and verify the accuracy of cohesion in the proposed method. The simulated P-CMOD curves are consistent with the previous experimental results, while the FPZ length and $K_{R}$ curves are different from the previous numerical results. The reasons that lead to the differences in results are discussed in detail. The comparative results in this paper will lead to a better insight, in which the accurate application of cohesive force on the FPZ has a crucial impact on the results of numerical simulations.

## 2. Methods and Materials

### 2.1. Nonlinear Spring Element

The user-defined nonlinear spring element of Ansys finite element software is used to complete the nonlinear finite element iterative solution process to achieve accurate cohesion applied on the concrete FPZ.

**Figure 2:**
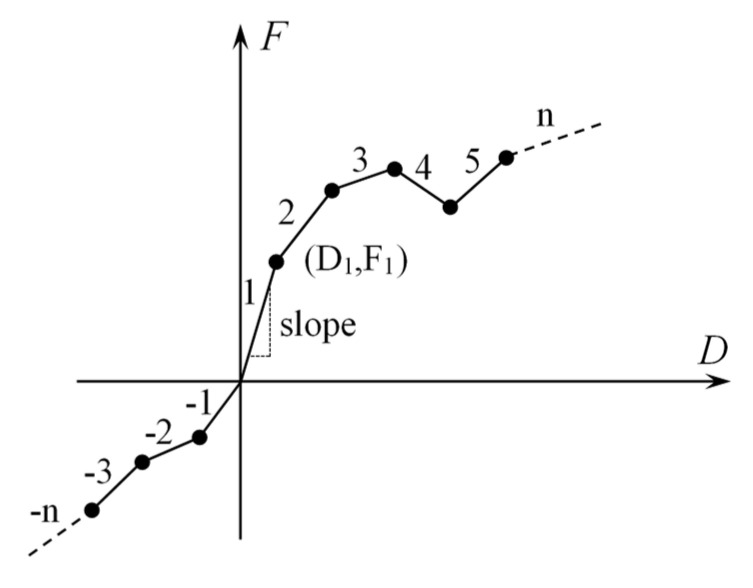
Generalized force-deflection curve of Combin39 element.

The unidirectional spring element with nonlinear generalized force deflection capability, combin39, can be utilized in the structural analysis [32]. This element can achieve longitudinal or torsional force in 1-D, 2-D, and 3-D application. In this paper, the element 1-D mode is used to apply the cohesive stress on the surface of a newly generated separate crack. The generalized force–deflection curve of this element is shown in Figure 2. The first and third quadrants indicate the tension and compression of the element, respectively. The element can output stress according to the user-defined force–deflection relationship curve.

**Figure 3:**
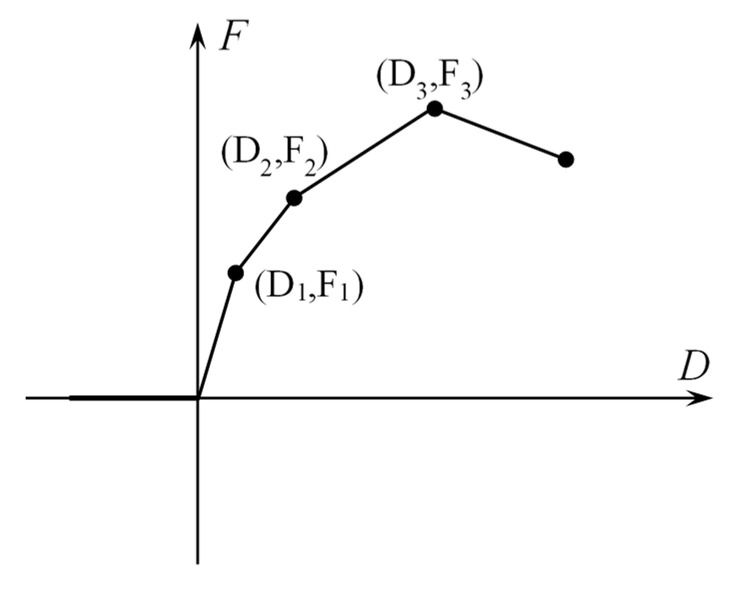
Combin39 force–deflection curves (KEYOPT(1) = 0, KEYOPT(2) = 1).

The Combin39 element controls the mechanical behavior through key options. KEYOPT(1) controls the unloading path of the element. Since there is no COD reduction in the FPZ of concrete mode I cracks, 0 is assigned to KEYOPT(1) to unload according to the F-D loading curve. KEYOPT(2) controls the deformation behavior of the element under compression. The Combin39 elements used in this simulation provide tensile force on the surface of the newly generated separate crack to simulate cohesion that hinders the crack from opening and expanding. The cohesion does not transmit compressive stress, for KEYOPT(2), and assigns a value of 1 so that the element does not provide compressive stress under compression, as shown in Figure 3. KEYOPT(3) = 0 makes the element provide stress along the *x*-axis direction of the element itself. KEYOPT(4) = 0 makes the element provide 1-D F-D relationships.

**Figure 4:**
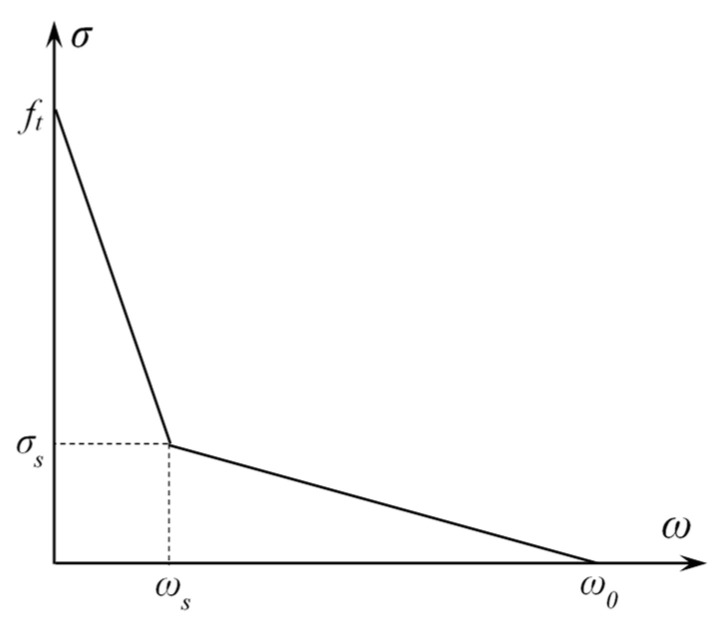
Petersson bilinear softening constitutive relationship.

A Combin39 element defines multiple points to represent the F-D relationship, so using this element can achieve stress softening behavior. The bilinear softening constitutive proposed by Petersson [11] is used in this paper to represent the relationship between cohesion and COD in the FPZ. This constitutive relationship is consistent with that used in previous research studies [21,22,23,24,25,26], so the results of this paper can be compared with those obtained before for discussion. The region under the softening constitutive curve represents the material’s fracture energy. The softening constitutive relationship is shown in Figure 4.

The corresponding formula is as follows:

(3) $$\sigma \left(w\right)=\{\begin{array}{c} {\;f}_{t}{-(f}_{t}{-\sigma }_{s}{)(w/w}_{s}),\;0\;\leq \;w\;\leq {\;w}_{s},\\ \sigma_{s}{(w}_{0}{-w)/(w}_{0}{-w}_{s}{),\;w}_{0}\;\leq \;w\;\leq {\;w}_{s}\\ \;0\;,\;\;\;\;\;w\;\geq {\;w}_{0}, \end{array},$$

where $f_{t}$ is the tensile strength of concrete, w is the opening displacement at any position between the newly generated crack surface, $w_{0}$ is critical crack opening displacement, $\sigma_{s}$ and $w_{s}$ separately represent the cohesive force and crack opening displacement of the turning point in a bilinear softening constitutive relationship. The concrete tensile strength $f_{t}$ and fracture energy $G_{f}$ determine $\sigma_{s}$, $w_{0}$, and $w_{s}$. The expressions are as follows:

(4) $$\sigma_{s}{=f}_{t}/3,$$

(5) $$w_{0}=3{.6G}_{f}{/f}_{t},$$

(6) $$w_{s}=0{.8G}_{f}{/f}_{t}.\;$$

**Figure 5:**
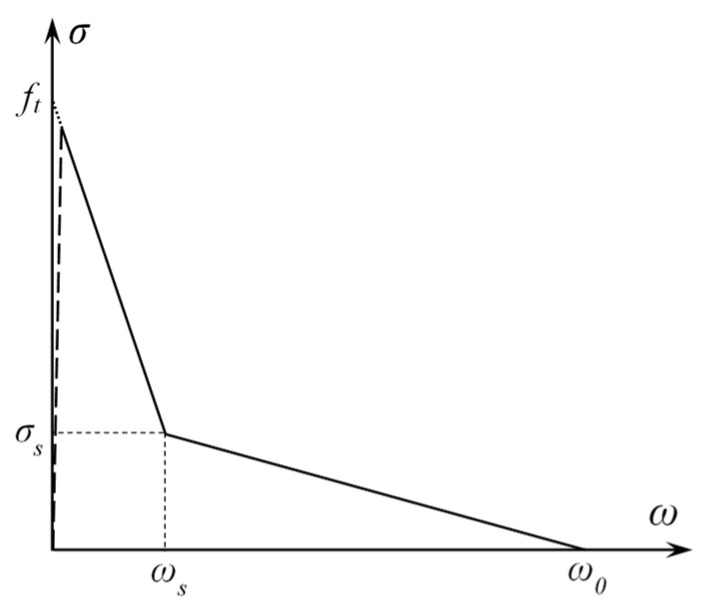
Combin39 constitutive relationship.

A point should be noted that the Combin39 element in Ansys requires the stiffness characterized by the first section in the first quadrant to be positive. To this end, a positive value must be assigned to the stiffness of the spring element. In addition, it is necessary to give the spring element an infinite initial stiffness, which is consistent with the softening constitutive relationship as in Figure 5. Therefore, the value corresponding to 0.1% of the critical crack opening displacement $w_{0}$ on the softening constitutive curve is used to calculate the initial stiffness. In fact, during the nonlinear solution process, the relative displacements of the nodes at both ends of the spring element with an initial length of zero will be greater than 0.1% $w_{0}$, and the effect of the first elastic section in the spring element constitutive relationship on the overall numerical simulation can be ignored.

### 2.2. Methods of Simulation

**Figure 6:**
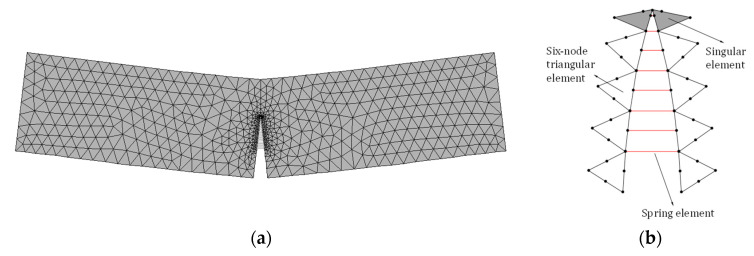
Schematic diagram of the finite element model: (**a**) the deformation of the model, (**b**) detailed introduction of the elements used in the model.

In this finite element analysis, the element in use is six-node triangular elements, the crack propagation length is set to 2 mm per step, and the gird near the crack needs to be encrypted. Different from the existing cohesive stress application method, after re-establishing and remeshing the model, the Combin39 nonlinear spring element should be added to connect the corresponding node on the crack surface, as shown in Figure 6. Due to the fine meshing of the model, a trapezoidal formula is used to apply nodal force at nodes of the triangular element instead of the nonuniform surface load of the element.

Then enter the solver, apply the load, and use the Newton–Raphson method to perform a nonlinear solution using line search. For nonlinear problems, Ansys uses Gaussian point results for calculation by default. At this time, the cohesive stress calculated is more accurate than the existing cohesion application method [21,22,23,24,25,26] under the same load constraint.

**Figure 7:**
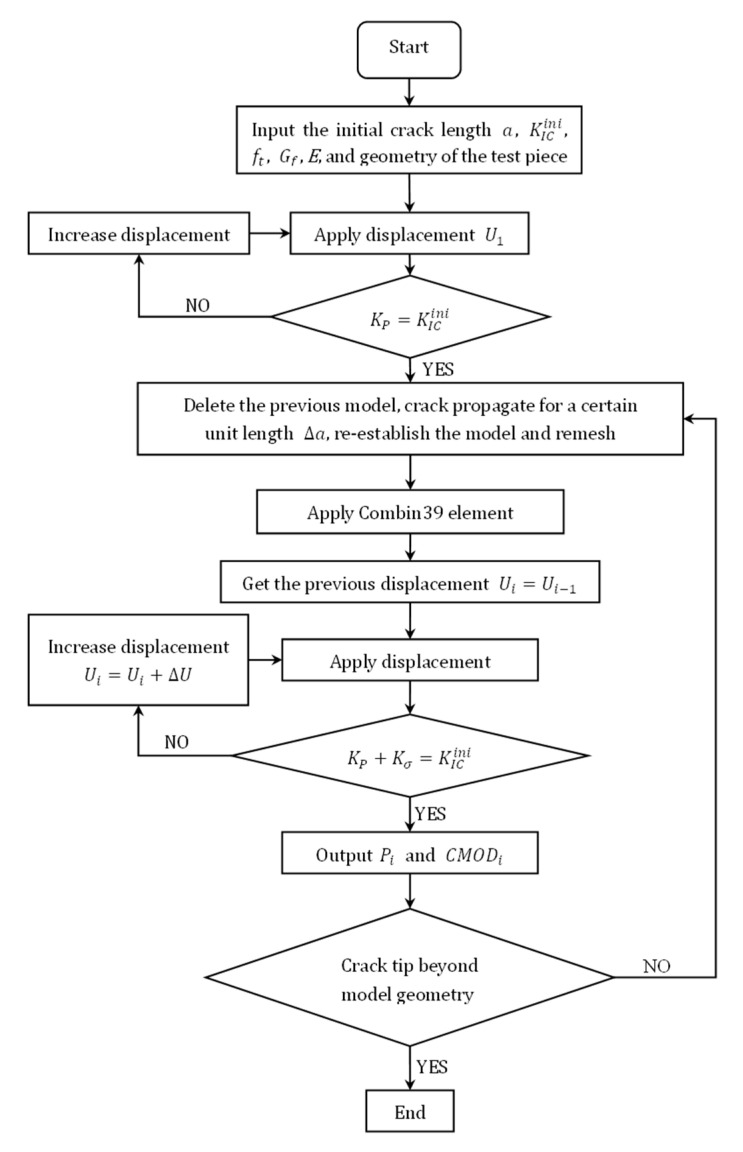
The improved crack propagations calculate the flow diagram.

Equation (2) is applied as the crack propagate criterion [21] in this paper. The entire crack propagation process numerical simulation algorithm is described as follows:

1. According to the test of geometric parameters and material parameters, a finite element model is established and meshed. The crack tip grid element should be performed to meet a singularity of −1/2.
2. Apply displacement constraints to the finite element model, and calculate the stress intensity at the crack tip. When it reaches the initial fracture toughness, the crack starts to propagate.
3. Crack spreads forward for a certain unit length Δa, and the finite element model is re-established and remeshed. Add the spring element between the newly generated interface.
4. Use the displacement results from the previous step as the initial displacement conditions for this step.
5. Determine whether the crack propagation criterion is satisfied. If not, increase the loading point displacement until the criterion is satisfied.
6. Repeat steps 4 to 5 until the crack tip extends to the edge of the model, and the simulation of the entire crack propagation process ends. The calculation flow diagram is shown in Figure 7.

In the simulation, different from the load control method used in previous literatures [21,22,23,24,25,26], a displacement control method consistent with the real test is used. In a practical test represented by a three-point bending beam test, displacement-controlled loading is used, and the external load at the loading point is measured by a load sensor. Compared with the load control, the displacement control can quickly get a convergent solution from the finite element as a boundary condition. In contrast, load-controlled loading will lose the calculation accuracy in the nonlinear calculation process, which requires more iterations and even does not converge. In this paper, the proposed spring element is used to apply cohesive stress on the concrete FPZ to simulate the mode I crack propagation process of three-point bending beams.

### 2.3. Materials for Simulation

**Figure 8:**
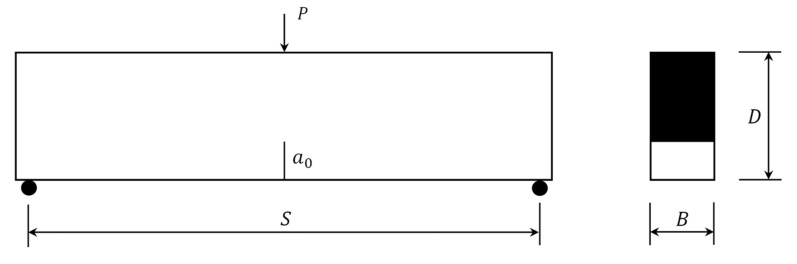
Notched concrete beam under three-point bending.

One set simulates concrete with different strengths, which is named C-series; the experimental data are from Dong [24] and Wang [33]; and concrete properties are shown in Table 1 (where $f_{c}$ represents the compressive strength, and E represents the elastic modulus). The size of the specimen in this set is S × D × B = 480 mm × 120 mm × 60 mm, and the initial crack ratio $a_{0}/D$ is set to 0.3, as shown in Figure 8.

Another set of data is from Dong [21]. This set is used to simulate beams with a different initial crack ratio $a_{0}/D$ and depth D. The specimens in the literature [21] were divided into two series, named B-series and L-series, and the same series names were still used in this paper. For the B-series, the size of specimens is retained at S × D × B = 600 mm × 150 mm × 40 mm, but the initial crack ratio $a_{0}/D$ varies from 0.2 to 0.6 (Table 2), and the material mechanical properties are $f_{t\;}$ = 2.4 MPa and E = 28 GPa. For L-series specimens, the span-to-depth ratio S/D is set to 4, the initial crack ratio $a_{0}/D$ is set to 0.4, the depth D varies from 100 mm to 300 mm (Table 3), and the material mechanical properties are $f_{t}\;$= 2.3 MPa and E = 24 GPa.

## 3. Results and Discussion

The simulation results of the crack propagation process based on the proposed method are compared with the previous experimental results and previously simulated results. In the same series, the crack propagation criterion, the softening constitutive, the material mechanical properties, and the specimen size are all the same except for the cohesion application method. In this section, the numerical method based on applying cohesion by the Combin39 nonlinear spring element is called iteration, and the previous numerical method that directly applies cohesion is called no iteration. The results will be discussed through four aspects: P-delta curve, P-CMOD curve, FPZ length, and $K_{R}$ curve.

### 3.1. P-Delta Curve

**Figure 9:**
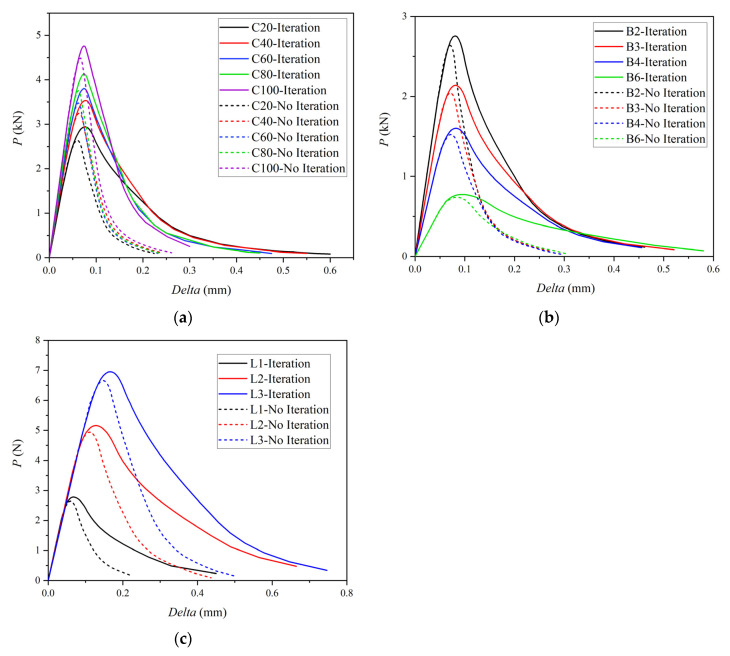
Three-point bending beam P-delta curves: (**a**) C20–C100, (**b**) B-series, (**c**) L-series.

The P-delta curves were not provided in the previous study, so the comparison between simulation results and experimental results cannot be performed. However, simulation results obtained from different methods can be compared. Fracture energy is represented by cohesion on the FPZ, and the fracture energy can be calculated by Equation (7):

(7) $$G_{f}=\frac{W_{0}+mg\delta_{0}}{A_{lig}},\;$$

where $W_{0}$ represents the external load work, which is equal to the enveloped area under the P-delta curve; $mg\delta_{0}$ represents the work done by specimen self-weight; and $A_{lig}=\left(D-a_{0}\right)\times B$ represents the ligament area. Therefore, fracture energy can be recalculated from the P-delta curves obtained by numerical simulation and compared with the input values to verify the accuracy of the applied cohesion with iteration. The P-delta curves obtained by the iterative and noniterative methods are plotted in Figure 9, and the plots enable us to find that the enveloped areas under the P-delta curves are significantly different under the two different methods. The fracture energy is recalculated according to Equation (7), and comparison results are shown in Table 4.

In Table 4, it can be concluded that the fracture energy calculated by the iterative method is close to the input values, while the fracture energy calculated by the noniterative method is much smaller than the input values. This conclusion is also consistent with the theoretical analysis, indicating that directly applying the cohesion approach results in a smaller value than the true cohesion value. In other words, the accuracy of cohesion can be improved by the proposed method, where cohesion is applied by the spring element.

### 3.2. P-CMOD Curve

**Figure 10:**
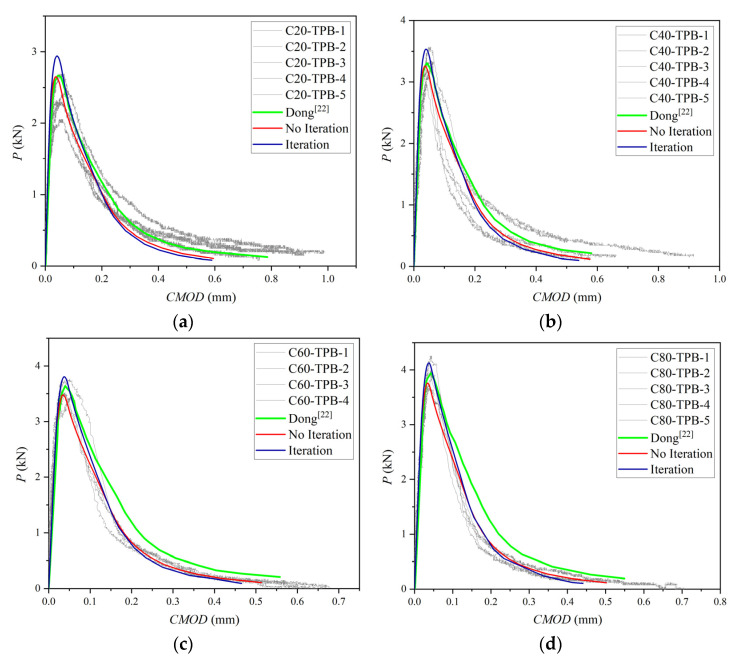

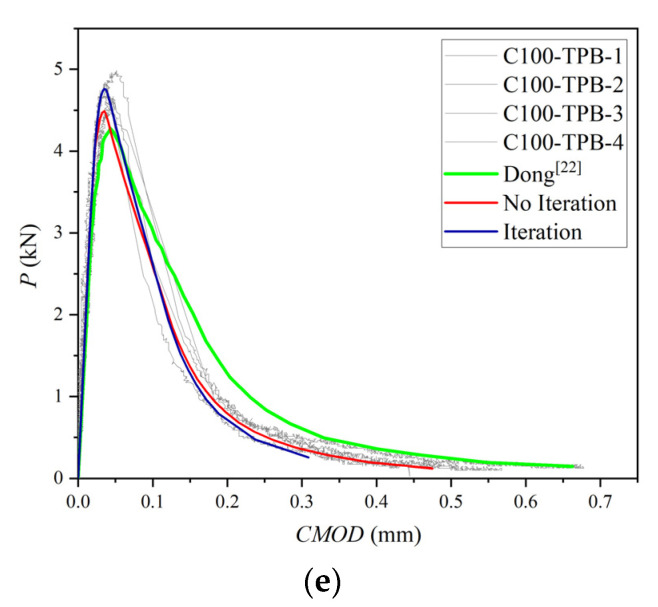
Three-point bending beam P-CMOD curves: (**a**) C20, (**b**) C40, (**c**) C60, (**d**) C80, (**e**) C100.

The test and simulated P-CMOD results of the C-series are plotted in Figure 10.

It is worth noting from Figure 10 that, except for the C20 group, the simulated curves by iteration fit the experimental curves perfectly. In each set of curves, the peak load of the simulation obtained by no iteration is less than that obtained by iteration. This is because without iteration, the COD result from external load is larger than the COD obtained from the superposition of external load and cohesion so that the corresponding cohesive force is less based on the softening constitutive. In contrast, the iteration method can get a larger but more accurate cohesion, so it can get a larger peak load according to the crack propagation criterion in use. The descending section of the curve obtained by the iteration method agrees better with test curves compared with the no iteration method. Previous simulation results [24] are plotted in Figure 10 in green. From the comparison of the results of the P-CMOD curves, it can be found that previous simulated curves given by Dong are close to the result of no iteration.

**Figure 11:**
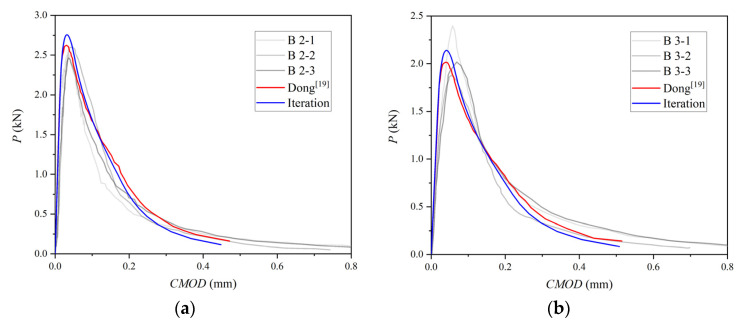

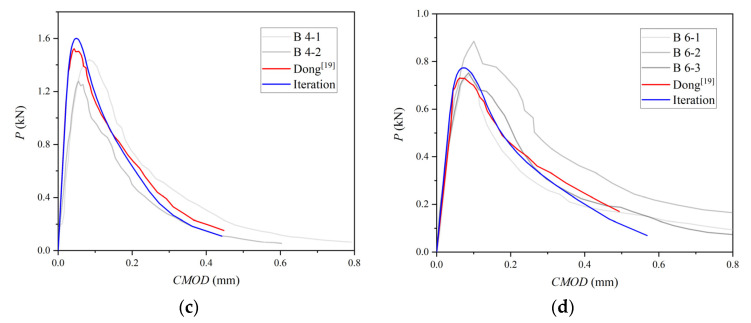
Three-point bending beam P-CMOD curves: (**a**) B2, (**b**) B3, (**c**) B4, (**d**) B6.

**Figure 12:**
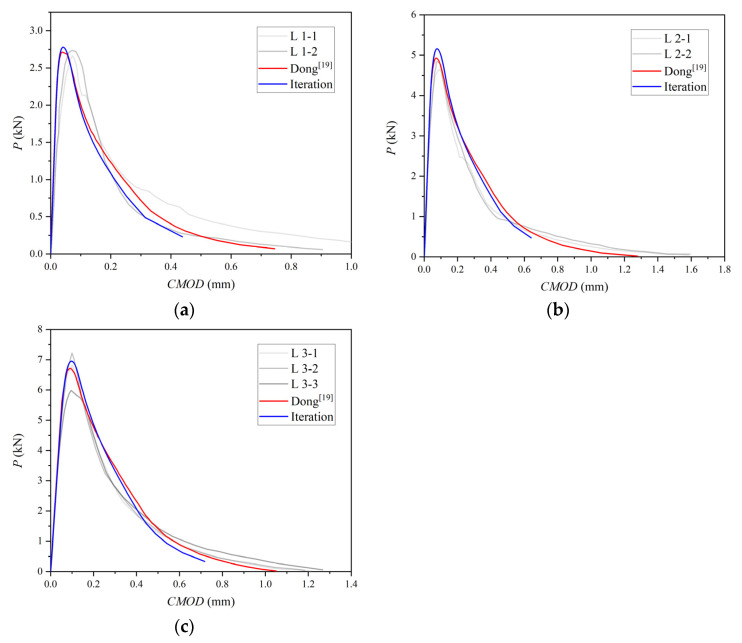
Three-point bending beam P-CMOD curves: (**a**) L1, (**b**) L2, (**c**) L3.

The experimental and simulated curves of the B-series and L-series are plotted in Figure 11 and Figure 12. P-CMOD curves of no iteration are consistent with previous simulated results, and only the P-CMOD curves from [21] are drawn in these figures for comparison.

The first linear segment in Figure 11 and Figure 12 represents the elastic deformation behavior of concrete. Using the point on the linear segment on the test curve, the elastic modulus of concrete can be calculated by Equation (8) [34]:

(8) $$\mathrm{CMOD}=\frac{24P\lambda }{\mathrm{EB}}\left[0.76-2.28\lambda +3.87\lambda^{2}-2.04\lambda^{3}+\frac{0.66}{{\left(1-\lambda \right)}^{2}}\right],$$

where λ is equal to ${(a+H}_{0}{)/(d+H}_{0})$, $H_{0}$ is the thickness of the knife edge holding the clip gauges used to measure CMOD and equal to 2 mm [24], P and CMOD are data value chosen on the linear segment in the test curve, and B is the thickness of the test specimen. The difference of the linear segment between the numerical simulation and the test indicates that the elastic modulus of the concrete measured by the test is not accurate enough. However, material parameters from Dong [21] are still used in the simulation for comparison. It can be seen from figures that the simulation has the same trend as the previous set.

Conclusions can be drawn that improving the accuracy of cohesion in a fictitious crack has a positive effect on the numerical simulation, and a curve obtained by the iterative method gives a larger peak load. The criterion based on material initial fracture toughness with iterative cohesion is suitable for simulating the P-CMOD curves of concrete with a different strength, $a_{0}/D$, and size.

### 3.3. FPZ Length

**Figure 13:**
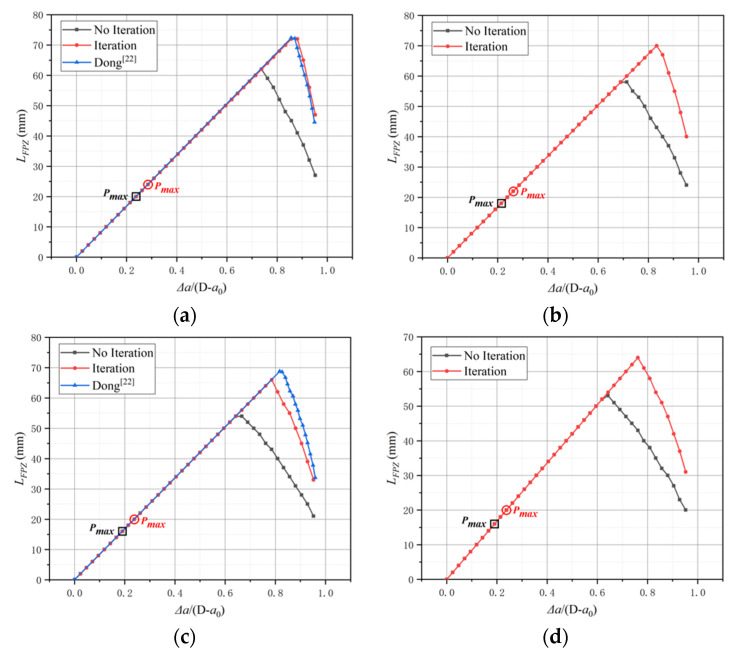

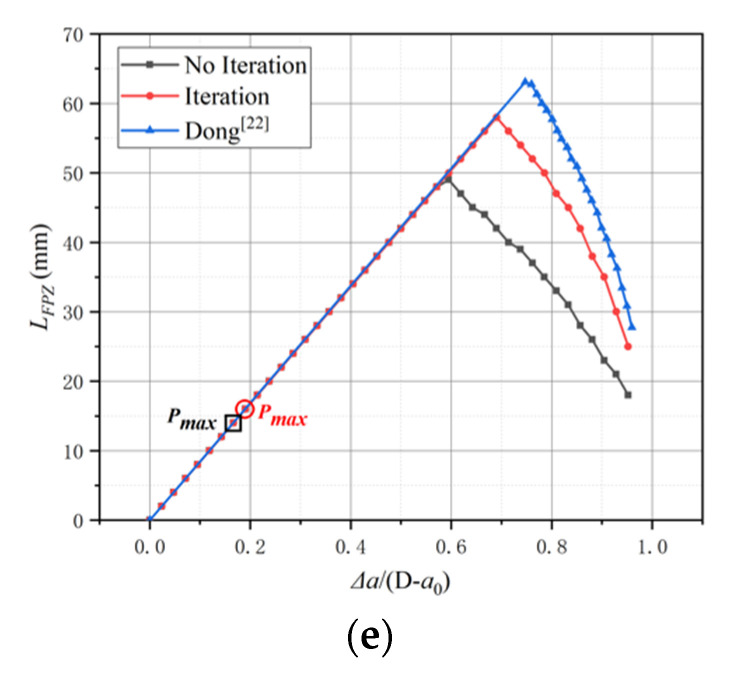
The variation in FPZ length: (**a**) C20, (**b**) C40, (**c**) C60, (**d**) C80, (**e**) C100.

**Figure 14:**
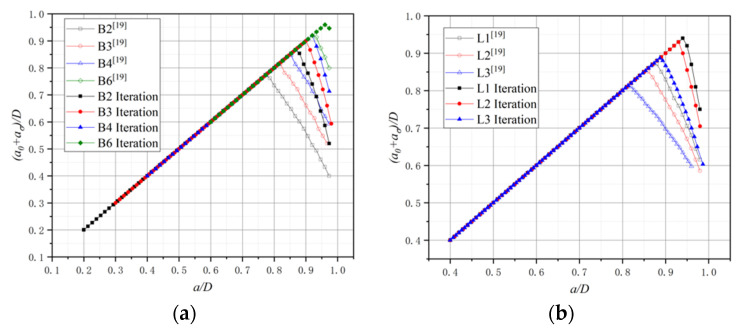
The variation in FPZ length: (**a**) B-series, (**b**) L-series.

The simulated FPZ length of the C-series as the crack propagation process is shown in Figure 13, where the horizontal axis is the ratio of the fictitious crack propagation length to the ligament length, expressed by ${\Delta a/(D\;-\;a}_{0})$. Only the C20, C60, and C100 FPZ length results are given by Dong [24]. For the B-series and L-series, the results are plotted in Figure 14, the horizontal axis represents the ratio of the crack length to the depth of the beam expressed by a/D, and the vertical axis represents the ratio of the sum of the initial crack length and FPZ length to the beam depth expressed by ${(a}_{0}{+a}_{\sigma })/D$.

The reason for choosing different coordinate systems in the comparison is to facilitate comparison with the original literature. From the comparison results in Figure 13, it can be found that after reaching the maximum load corresponding to the P-CMOD curve, the FPZ length simulated by a different method keeps growing with the crack propagation. The iteration method obtains a greater maximum length value compared with the no iteration method, as shown in Figure 13 and Figure 14. The cohesion will appear at the region with COD less than $w_{0}$ after multiple iterations, while cohesion will not exist at a part of the same region whose COD is greater than the critical value without iteration. After FPZ hits the maximum value, the FPZ length with iteration decreases more significantly. The numerical simulated results simulated by Dong et al. [21,24] were compared with the numerical simulated results in this paper. The variation of the C20 group is close to the iteration result, but the FPZ length of the C60 and C100 groups exceeds the iteration results. It may suggest that a certain iterative process during simulation process, “repeatedly solving COD and σ”, is conducted when calculating the FPZ length. However, the results are unstable and not accurate enough for the C-series.

For the B-series and L-series, FPZ lengths obtained from Dong et al. [21] are smaller than the results of the iterative ones. In the process of the iteration method, after adding the Combin39 nonlinear spring element, only boundary conditions of the model are needed. The load P, COD, and σ are solutions of nonlinear equations so that the cohesion applied in the FPZ can be characterized more accurately by the iteration method. Next, factors that affect the FPZ length based on the iterative results are figured out as follows.

For the C-series, it is found that with the increase in concrete strength, the maximum FPZ length decreases. Based on the softening law, fracture energy and tensile strength are the main factors in determining critical crack opening displacement. Small critical crack opening displacement results in small FPZ length. The fracture energy for the C-series is close, so the concrete with higher tensile strength results in a shorter length of the FPZ. For the B-series, as $a_{0}/D$ decreases, the maximum FPZ length increases since the beam with smaller $a_{0}/D$ has a longer ligament length, which can make the FPZ fully develop. For the L-series, as the depth of the beam increases, the maximum FPZ length increases, and the principle is the same as the B-series. From another perspective, it can be concluded that the geometry shape of beams affects the FPZ length.

### 3.4. $K_{R}$ Curve

**Figure 15:**
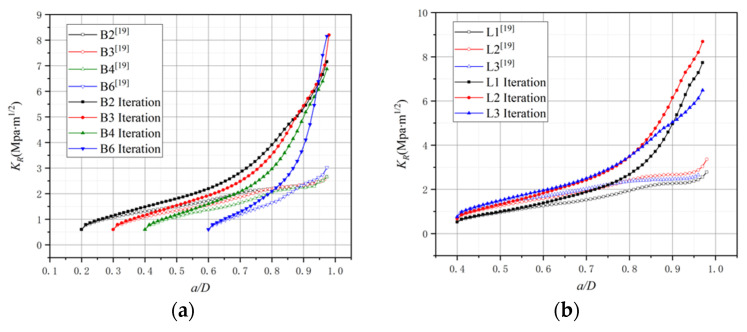
The $K_{R}$ curves: (**a**) B-series. (**b**) L-series.

The $K_{R}$ curves are used to represent the change in fracture toughness in the process of crack propagation. Foote et al. [35] proposed a theoretical analysis model of the $K_{R}$ curve before the FPZ fully developed. The theoretical model for analyzing the $K_{R}$ curve proposed by Hu and Wittmann [36] accurately restored the wedge opening loaded mortar specimens’ test results. Xu and Reinhardt [37] proposed a $K_{R}$ calculation model based on cohesion in the FPZ where $K_{R}$ was the stress intensity factor generated by initial fracture toughness $K_{\mathrm{IC}}^{\mathrm{ini}}$ and cohesion in the FPZ together. However, an assumption was made that the FPZ length and the cohesive stress distribution stayed unchanged after the FPZ fully developed. Therefore, the $K_{R}$ curve increased as the ratio of the effective crack length to the beam depth increased. Lutz and Swain [38,39] found that the $K_{R}$ resistance curve for ceramic brittle materials increased as the increase in the FPZ length. After the FPZ hit the maximum value, $K_{R}$ remained a stable value. This result was consistent with the conclusion explored by Xu et al. [40]. Dong et al. [21] proposed another method for numerically calculating the $K_{R}$ curve, in which $K_{R}$ is calculated by:

(9) $$K_{R}\left(\Delta a\right){=K}_{\mathrm{Ic}}^{\mathrm{ini}}{+K}_{I}^{\sigma }{=K}_{I}^{P}\left(P,\Delta a\right),$$

where Δa means the crack propagation length in numerical simulation, and $K_{R}\left(\Delta a\right)$ and $K_{I}^{P}\left(P,\Delta a\right)$ represent the stress intensity factor of the crack extension resistance and external load, respectively. In other words, $K_{R}$ is equivalent to the stress intensity factor corresponding to the external load during crack propagation. This paper uses the proposed spring element model to calculate $K_{R}$ curves based on Equation (9). The simulated $K_{R}$ curves’ results of the B-series and L-series from this paper and previous simulation results [21] are plotted in Figure 15 for comparison.

**Figure 16:**
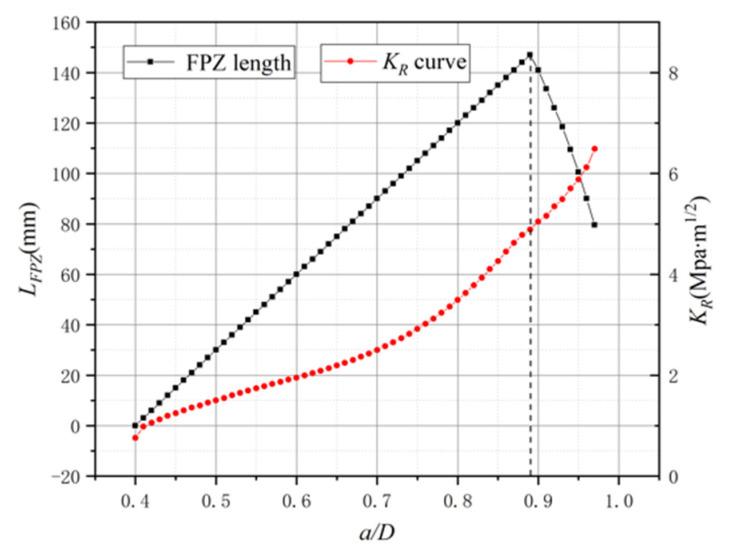
The FPZ length and $K_{R}$ curve of L3.

In the conclusion of a previous research [21], there was a plateau in the $K_{R}$ curve with the FPZ length variation. However, it can be found that there is no plateau existing in the $K_{R}$ curve by iterative approach in Figure 15. In comparison with Figure 13 and Figure 14, the $K_{R}$ curve still exhibits an increasing trend after the FPZ length has fully developed, and only the raising rate becomes slightly decreased. To further explore the relationship between the FPZ length and the $K_{R}$ curve, this paper draws the FPZ length and $K_{R}$ curve of the L3 specimen together in Figure 16. From Figure 16, it can still be inferred that the FPZ length affects the shape of the $K_{R}$ curve. After the FPZ length has fully developed, the $K_{R}$ curve growth rate decreases. The value of the $K_{R}$ curve simulated in this paper is larger than that from previous simulations [21], and at the end of the curves, the ratio of the $K_{R}$ values obtained by two methods is over two times. The difference in results verifies that it is essential to apply cohesive force in FPZ considering iteration.

It is necessary to reiterate that for the B-series and L-series, each set shares the same material mechanical parameter except for $G_{f}$ and $K_{\mathrm{Ic}}^{\mathrm{ini}}$. It is more appropriate to explore the factors that affect the $K_{R}$ curve from these two series first. According to Figure 14b and Figure 15b and Table 3, it can be found that the fracture energy $G_{f}$ affects the shape of the $K_{R}$ curve. Although the depths of beams are different from the L-series, the FPZ length variation of the L1 and L2 specimens is similar from overall view in Figure 15b. The reason why the $K_{R}$ curve value of L2 is greater than L1 is that $G_{f}$ of L2 is larger than that of L1. Comparing the $K_{R}$ curve of L1 with L3, though $G_{f}$ of L3 is greater than that of L1, the FPZ length variation is the main reason for L3 getting a smaller $K_{R}$ value at the end of the curve at this moment. For the B-series as in Figure 14a, the initial crack ratio $a_{0}/D$ of specimens is different so the $K_{R}$ curve starts at different points, but at the end of the curve, $G_{f}$ is the main reason for the difference in the $K_{R}$ value.

**Figure 17:**
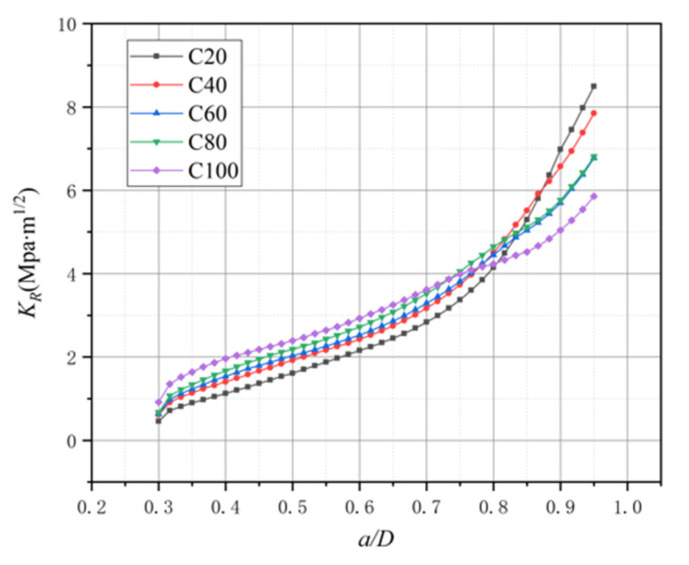
The $K_{R}$ curve of the C-series.

The $K_{R}$ curves of the C-series are plotted in Figure 17. The $K_{R}$ value of higher-strength concrete is the highest at first, but then it becomes the smallest. It seems like the tensile strength affects the $K_{R}$ value, and the concrete with a higher tensile strength will get a lower $K_{R}$ value at the end of the curve. According to the data in Table 1, it is found that the fracture energy of concrete with different strengths obtained from the experiment is relatively close. However, as discussed above about the influencing factors for the FPZ length of the C-series, higher tensile strength results in smaller maximum FPZ length under the same fracture energy. Therefore, it can be concluded that the FPZ length is the main reason for the variation in the $K_{R}$ value of the C-series.

## 4. Conclusions

Aiming at the numerical simulation of the concrete mode I crack propagation process, a numerical method using the Combin39 nonlinear spring element to apply cohesive stress on the FPZ is proposed in this paper. Based on displacement control, the values of cohesion on the FPZ are nonlinear solutions according to the Newton–Raphson method using line search. According to the comparison between the above numerical calculation results and test results, conclusions are drawn as follows:

1. The P-delta curves obtained from numerical simulation were used to recalculate fracture energy and compared with the input value. The direct application of cohesion produced results that were much smaller than the true value, while the proposed method of applying cohesion by the spring element can improve the accuracy of the applied cohesion.
2. Using the proposed method to apply the cohesive stress on the FPZ for numerical calculation, though the peak load obtained is larger than the no-iteration result, P-CMOD curves obtained are still fitted the experimental results, indicating that the cohesion is applied in a reasonable manner, and the crack propagation criterion based on material initial fracture toughness is suitable for numerical simulation.
3. The FPZ length obtained by the iteration method can reach a larger maximum value compared with no-iteration, and the decline gradient of the FPZ length also becomes larger after the FPZ is fully developed. The fracture energy, tensile strength, and geometry shape of the beam are main reasons for deciding the FPZ length. According to the bilinear softening constitutive, fracture energy and tensile strength determine the critical crack opening displacement. A larger critical crack opening displacement or a longer ligament makes it easier to obtain a larger FPZ length.
4. The $K_{R}$ curve obtained with iteration is significantly different from the noniterative curve. The $K_{R}$ value is continually rising with the crack propagation. After the FPZ length has fully developed, the rising rate of the $K_{R}$ curve has become slow. Through the synergistic comparison with the change of the FPZ length, it is proved that the $K_{R}$ value is mainly affected by the fracture energy and FPZ length. The iterative method is more suitable for simulating the $K_{R}$ curves by improving the accuracy of the cohesion on the FPZ.

The significant difference in numerical results indicates that applying cohesion is essential for numerical simulation, and the accuracy of cohesion has a great influence on the results of numerical simulation. The method has been proved to be well applicable to numerical simulation for the concrete mode I crack propagation process by improving the accuracy of cohesion applied on concrete FPZ. According to the crack propagation criterion based on initial fracture toughness, more numerical simulations are needed to explore the effect of the cohesion application in the modes I–II crack propagation process, which is one of our future studies.

## Figures and Tables

**Table 1:** Material properties.

Concrete	$f_{c}$ (MPa)	$f_{t}$ (MPa)	E (GPa)	$K_{\mathrm{Ic}}^{\mathrm{ini}}$ $(\mathrm{MPa}\cdot m^{1/2})$	$G_{f}$ (N/m)
C20	32.8	3.05	29.9	0.461	117.1
C40	48.9	3.74	33.2	0.616	124.5
C60	69.9	4.43	35.7	0.632	114.9
C80	84.1	5.01	38.1	0.667	120.5
C100	115.8	5.71	41.4	0.917	115.4

**Table 2:** Size and material parameters for B-series beams.

Specimen	S × D × B(mm)	$a_{0}/D$	$K_{\mathrm{IC}}^{\mathrm{ini}}$ $(\mathrm{MPa}\cdot m^{1/2})$	$G_{f}$ (N/m)
B2-1	600×150×40	0.2	0.60	96
B2-2			0.59	100
B2-3			0.62	92
Avg.			0.60	96
B3-1	600×150×40	0.3	0.65	100
B3-2			0.63	105
B3-3			0.53	88
Avg.			0.60	98
B4-2	600×150×40	0.4	0.58	85
B4-3			0.61	100
Avg.			0.60	92.5
B6-1	600×150×40	0.6	0.60	105
B6-2			0.62	120
B6-3			0.58	100
Avg.			0.60	108

**Table 3:** Size and material parameters for L-series beams.

Specimen	S × D × B(mm)	$a_{0}/D$	$K_{\mathrm{Ic}}^{\mathrm{ini}}$ $(\mathrm{MPa}\cdot m^{1/2})$	$G_{f}$ (N/m)
L1-1	400×100×100	0.4	0.52	104
L1-2			0.52	90
Avg.			0.52	97
L2-1	800×200×100	0.4	0.71	155
L2-2			0.62	151
Avg.			0.67	153
L3-1	1200×300×100	0.4	0.78	123
L3-2			0.75	146
L3-3			0.75	153
Avg.			0.76	141

**Table 4:** Recalculated fracture energy comparison table.

Group	mg (N)	$\delta_{0}$ (mm)	$W_{0}$ (N·mm)	$A_{lig}$ (mm × mm)	$G_{f}-\mathrm{Recalc}$ (N/m)	$G_{f}$ (N/m)	%
C20-Iteration	81.37	0.60	538.5	84 × 60	116.5	117.1	−0.5%
C20-No Iteration		0.22	224.2		48.0		−59.0%
C40-Iteration	81.37	0.55	592.5	84 × 60	126.4	124.5	1.5%
C40-No Iteration		0.24	286.0		60.6		−51.3%
C60-Iteration	81.37	0.48	553.0	84 × 60	117.5	114.9	2.2%
C60-No Iteration		0.23	283.5		60.0		−47.8%
C80-Iteration	81.37	0.45	581.3	84 × 60	122.6	120.5	1.7%
C80-No Iteration		0.23	303.0		63.8		−47.0%
C100-Iteration	81.37	0.30	571.6	84 × 60	118.3	115.4	2.5%
C100-No Iteration		0.26	374.4		78.5		−32.0%
B2-Iteration	84.76	0.46	438.2	120 × 40	99.5	96.0	3.6%
B2-No Iteration		0.26	235.8		53.7		−44.0%
B3-Iteration	84.76	0.52	385.9	105 × 40	102.4	98.0	4.5%
B3-No Iteration		0.27	203.2		53.8		−45.1%
B4-Iteration	84.76	0.46	300.2	90 × 40	94.1	92.5	1.8%
B4-No Iteration		0.29	164.3		52.5		−43.3%
B6-Iteration	84.76	0.48	207.1	60 × 40	106.8	108.0	−1.1%
B6-No Iteration		0.30	104.9		54.3		−49.7%
L1-Iteration	94.18	0.45	523.0	60 × 100	94.2	97.0	−2.9%
L1-No Iteration		0.22	256.7		46.2		−52.3%
L2-Iteration	376.7	0.66	1555.2	120 × 100	150.3	153.0	−1.8%
L2-No Iteration		0.44	831.3		82.1		−45.7%
L3-Iteration	847.6	0.75	2178.4	180 × 100	156.2	141.0	10.8%
L3-No Iteration		0.50	1308.6		96.2		−31.7%

* $G_{f}$-recalc means recalculated fracture energy; % means the difference percentage of $G_{f}$-recalc from the material’s $G_{f}$.

## Data Availability

The data presented in this study are available on request from the corresponding author.
